# Impact of APOE-ε alleles on brain structure and cognitive function in healthy older adults: A VBM and DTI replication study

**DOI:** 10.1371/journal.pone.0292576

**Published:** 2024-04-18

**Authors:** Colleen Lacey, Theone Paterson, Jodie R. Gawryluk, for the Alzheimer’s Disease Neuroimaging Initiative

**Affiliations:** 1 Department of Psychology, University of Victoria, Victoria, British Columbia, Canada; 2 Institute on Aging and Lifelong Health, University of Victoria, Victoria, British Columbia, Canada; 3 Division of Medical Sciences, University of Victoria, Victoria, British Columbia, Canada; Georgetown University Medical Center, UNITED STATES

## Abstract

**Background:**

The Apolipoprotein E (APOE) gene has been established in the Alzheimer’s disease (AD) literature to impact brain structure and function and may also show congruent effects in healthy older adults, although findings in this population are much less consistent. The current study aimed to replicate and expand the multimodal approach employed by Honea et al. Structural magnetic resonance imaging (MRI), diffusion tensor imaging (DTI), and neuropsychological measures were used to investigate the impact of APOE-ε status on grey matter structure, white matter integrity, and cognitive functioning.

**Methods:**

Data were obtained from the Alzheimer’s Disease Initiative Phase 3 (ADNI3) database. Baseline MRI, DTI and cognitive composite scores for memory (ADNI-Mem) and executive function (ADNI-EF) were acquired from 116 healthy controls. Participants were grouped according to APOE allele presence (APOE-ε2+ N = 17, APOE-ε3ε3 N = 64, APOE-ε4+ N = 35). Voxel-based morphometry (VBM) and tract-based spatial statistics (TBSS) were used to compare grey matter volume (GMV) and white matter integrity, respectively, between APOE-ε2+ and APOE-ε3ε3 controls, and again between APOE-ε4+ and APOE-ε3ε3 controls. Multivariate analysis of covariance (MANCOVA) was used to examine the effects of APOE polymorphism on memory and EF across all APOE groups with age, sex and education as regressors of no interest. Cognitive scores were correlated (Pearson r) with imaging metrics within groups.

**Results:**

No significant differences were seen across groups, within groups in MRI metrics, or cognitive performance (p>0.05, corrected for multiple comparisons).

**Conclusions:**

The current study partially replicated and extended previous findings from an earlier multimodal study (Honea 2009). Future studies should clarify APOE mechanisms in healthy ageing by adding other imaging, cognitive, and lifestyle metrics and longitudinal design in larger sample sizes.

## Introduction

The Apolipoprotein E (APOE) gene has been well established in the Alzheimer’s disease (AD) literature to impact brain structure and function and modulate the risk for AD. APOE mediates lipid transport, and different isoforms are thought to affect Aβ aggregation and clearance differently [1]. The three isoforms, ε2, ε3 and ε4, occur at global frequencies of 8.4%, 77.9% and 13.7% respectively, although the ε4 allele is present in approximately 40% of individuals with late-onset AD [2]. Strikingly, the odds of developing AD are approximately 14 times higher in individuals with two ε4 alleles and ten times higher than in individuals with ε3ε4; thus, APOE-ε4 is considered an important biomarker in predicting whether or not an individual will go on to develop late-onset AD [3, 4]. In contrast, the ε3 allele has been proposed to play a neutral role in predicting cognitive ageing (i.e., neither increasing nor decreasing the likelihood of developing AD and the ε2-allele has been proposed to play a protective role, with the odds of developing AD reduced in individuals with ε2ε2 and ε2ε3 [5].

Research to date has predominantly focused on the APOE-ε4 allele and neurodegeneration. Evidence from post-mortem cellular research shows that APOE-ε4 disrupts cholesterol and oligodendrocyte functioning, leading to problems with the myelination process [6]. In-vivo Magnetic resonance imaging (MRI) studies investigating the impact of the APOE-ε4 allele on the healthy aging brain have posited widespread white matter integrity (WMI) and grey matter volume (GMV) changes. However, findings on the impact of APOE-ε status on brain structure have been mixed. Some studies have shown cortical thinning and reduced GMV in regions such as the superior temporal gyrus and hippocampus in healthy APOE-ε4 carriers compared to non-ε4 carriers [7–11]. Others have found WMI reduction, without GMV changes, in healthy APOE-ε4 carriers compared to non-ε4 carriers [12, 13].

A notable study by Honea et al. investigated the effect of APOE-ε4 on brain structure and cognitive function using multimodal imaging methods in healthy older adults [7]. While they found no differences in WMI or GMV after correcting for multiple comparisons, they found interesting trends, with differences in medial temporal GMV and WMI between carriers and non-carriers of the ε4-allele. Alongside MRI metric differences, Honea et al. found cognitive performance differences between healthy older carriers of the ε4-allele and non-carriers, specifically reporting reduced performance on memory and working memory measures in APOE-ε4 carriers [7].

Taken together, these studies suggest that APOE-ε4 may lead to structural differences in the healthy aging brain. However, further replication and characterization of the impact of APOE-ε4 status in the healthy older population is paramount. Particularly, questions remain on whether reliable changes occur to both WMI and GMV, where they are localized, and how they relate to cognitive functioning.

In addition to studies focused on replicating of APOE-ε4 imaging findings, there is a need to examine how other APOE genotypes might influence brain structure in healthy older adults. In particular, less attention has been given to ε2-carriers despite evidence that the APOE-ε2 allele may be neuroprotective against AD [5]. Research that has included APOE ε2-carriers has also revealed mixed findings, with some studies indicating preserved structural integrity of grey matter and increased WMI in these individuals [14], and others not finding protective effects of ε2 in the brain, suggesting that ε2 may play a different role in healthy ageing that has yet to be sufficiently explained [15, 16]. Evidently, the effects of APOE-ε2 are much less consistent and are understudied relative to APOE-ε4.

In order to replicate and extend research on APOE in healthy ageing, the current study aimed to reproduce Honea et al.’s study, given that their approach was multimodal and investigated GMV, WMI, and cognitive function in APOEε4 carriers relative to ε3ε3 controls [7]. Using data from the healthy cohort from the Alzheimer’s Disease Neuroimaging Initiative (ADNI), the current study also aimed to expand Honea et al.’s methods to include an APOE- ε2 group, as well as cognitive composites for memory and executive function [7]. It was hypothesized that there would be structural differences in WMI and GMV across APOE genotypes (i.e., APOE ε4-carriers < ε3ε3 controls < ε2-carriers) in a cohort of cognitively healthy older adults in temporal, prefrontal, and hippocampal regions. It was also hypothesized that similar cognitive differences would be seen across the current study’s APOE groups in the specific domains of memory and executive function, given that composite measures for these domains were available within the ADNI and have shown good reliability across several ADNI studies.

## Methods

### Participants

Data used in the current study were downloaded from the Alzheimer’s Disease Neuroimaging Initiative (ADNI) database between December 2020 and April 2021. The ADNI is a large multi-center research trial launched in 2003 by the National Institute on Ageing (NIA), the National Institute of Biomedical Imaging and Bioengineering (NIBB), the Food and Drug Administration (FDA), private pharmaceutical companies and non-profit organizations, as a $60 million, 5-year public-private partnership. The primary goal of ADNI has been to investigate whether serial MRI, positron emission tomography, other biological markers, and clinical neuropsychological assessments can be combined to measure the progression of mild cognitive impairment (MCI) and AD. Please see www.adni-info.org for up-to-date information. The current study exclusively examined data available in the ADNI3 protocol, which began in 2016 with an expanded goal of determining clinical, cognitive, imaging, genetics, and biochemical biomarker characteristics across AD (including 133 new elderly control participants) [17].

Data obtained from ADNI3 included 317 healthy older adults with both baseline DTI and MRI imaging data (controlled for scanner and image acquisition type), composite cognitive scores for memory and EF, and APOE genotyping. Participants were included in the healthy cognitive group if they were free of subjective memory concerns, scored within the normal range on the Wechsler Memory Scale-Revised (WMS-R) Logical Memory II adjusted for age/education (≥ 9 for those with ≥ 16 years of education), scored between 24 and 30 on the Mini Mental State Examination (MMSE), had a Clinical Dementia Rating (CDR) of 0, and had no other impairments to cognition or activities of daily living. For more eligibility information, please see the ADNI3 Procedures Manual Chapter 6 (https://adni.loni.usc.edu/wp-content/uploads/2012/10/ADNI3-Procedures-Manual_v3.0_20170627.pdf). Participants were excluded if scans were distorted and unsuitable for image preprocessing. The final sample size after applying exclusion criteria was 116. The sample size was determined based on previous neuroimaging studies that have shown significant structural differences between healthy older APOE groups [13, 18–23]. A separate power analysis was conducted for cognitive analyses (MANCOVA) via G*Power software (version 3.1.9.6), which suggested a total sample size of 153 [24, 25].

Cognitively healthy older adults were assigned to three groups based on APOE genotype: (i) APOE ε4+ (including individuals with ε3ε4 and ε4ε4), (ii) APOE ε3ε3 (controls), and (iii) APOE ε2+ (including individuals with ε2ε3 and ε2ε2). Those with ε2ε4 were excluded due to qualifying for inclusion into more than one group (i.e., both an ε2 and ε4 carrier) or if no APOE data was reported in ADNI3. APOE groups were matched for age, sex, and education. Demographic and cognitive data are summarized in Table 1. Twenty-six participants had a known family history of AD (N_ε2+_ = 3, N_ε3ε3_ = 12, N_ε4+_ = 11). Twenty-five individuals were excluded in the preprocessing steps for corrupted image quality, including severe image artifacts and cut-off cortical structures, with a final sample of 116 (N_ε2+_ = 17, N_ε3ε3_ = 64, N_ε4+_ = 35). Of note, all e2e2 participants were excluded from screening and preprocessing. An additional 9 ε3ε3 participants were excluded in VBM only due to artifacts to T1 images. This did not affect the significance across group demographics overall.

**Table 1:** Demographic information and cognitive data for APOE groups.

	APOE ε2+	APOE ε3 ε3	APOE ε4+	F/X^2^	p-value
**N**	17 (17 ε2ε3/0 ε2ε2)	64	35 (28 ε3ε4/7 ε4ε4)	—	—
**Age (M/SD)**	77.31/16.54	74.31/8.69	73.17/7.95	1.42	0.25
**Sex (F/M)**	8/9	42/22	21/14	1.98	0.37
**Education (M/SD)**	17.00/2.65	16.84/2.28	16.74/2.21	0.07	0.93
**AD Family History (Y/N)**	3/14	12/52	11/24	2.35	0.31
**ADNI-Mem (M/SD)**	0.78/0.66	1.11/0.54	1.00/0.66	1.85	0.16
**ADNI-EF (M/SD)**	0.98/0.49	0.98/0.91	1.09/0.91	0.20	0.83

*Note*. *M* and *SD* represent mean and standard deviation, respectively. *F* statistics with p-values reported from ANOVA, *X*^2^ statistic with p-value reported for categorical sex variable.

### Ethics statement

ADNI’s primary investigators obtained written informed consent for all participants according to each imaging and assessment site’s Institutional Review Board. Data obtained was de-identified to protect the participant’s confidentiality, and the authors of the current study did not have access to identifying participant information during data collection. Researchers using data from the ADNI database must apply to obtain access and sign the *ADNI Data Use Agreement* (for more details, please visit https://adni.loni.usc.edu/wp-content/uploads/how_to_apply/ADNI_Data_Use_Agreement.pdf), which was granted for the current study and updated again in May of 2021. Further, the current study was approved by the University of Victoria’s Human Research Ethics Board (HREB, no. 14–083, 22–0043).

### Study design

First, the ε4+ group was compared to the ε3ε3 controls, and the ε2+ group was compared to the ε3ε3 group on VBM and DTI to assess grey matter and white matter structure, respectively, in the FMRIB Software Library (FSL). Next, cognitive scores were compared to assess brain function using multivariate analysis of covariance (MANCOVA). Finally, within-group cognitive neural correlates were assessed and compared across the three groups by extracting imaging metrics and correlating them with cognitive composite scores within each APOE group.

### Data acquisition and preprocessing

#### APOE Genotyping

All APOE genotyping was performed upon baseline visit and was conducted by collecting DNA samples obtained from participants’ blood [26]. Genetic samples were collected in 10mL Purple ethylenediaminetetraacetic acid (EDTA) blood collection tubes and then analyzed at the National Centralized Repository for Alzheimer’s Disease (NCRAD) labs. More details on genotyping protocols and toolkit information can be found in the ADNI3 protocol, Chapter 21 (https://adni.loni.usc.edu/wp-content/uploads/2012/10/ADNI3-Procedures-Manual_v3.0_20170627.pdf).

#### Voxel-based morphometry

Grey matter volumetric characteristics were evaluated using VBM [27, 28]. 3T Accelerated Sagittal T1-weighted magnetization-prepared rapid gradient echo (MPRAGE) scans were downloaded from ADNI3 for each participant. The following acquisition parameters were set for each participant: PA Matrix coil, a repetition time (TR) of 2300 ms, an echo time (TE) of 3.0 ms, an inversion time (TI) of 900 ms, a flip angle of 9.0 degrees, pulse sequence GR/IR, Matrix X with 240 pixels, matrix Y with 256 pixels, matrix Z with 160 pixels, pixel spacing of 1.1 mm, and a slice thickness of 1.2 mm.

Data preprocessing was carried out using FSL (version 5.1.10). For VBM preprocessing, images were converted to an image file type compatible with the FSL program (*NIfTI* format) using MRICRON program dcm2nii. Scans for each participant were visually inspected for any major artifacts or distortion, and each participant’s brain was extracted from the skull and neck, adjusting fractional intensity thresholds to account for individual differences (BET; [29]). Next, images were segmented into grey matter, white matter, and CSF, where the grey matter from each subject was then registered to standard space (i.e., MNI 152) with non-linear registration. Images were averaged to create a study-specific grey matter template and represented as a 4D image. Each grey matter image was registered to the study-specific template, modulated, and smoothed with a kernel of sigma = 3mm. Finally, Randomise permutation testing with threshold-free cluster enhancement (TFCE) was computed to compare GMV differences between APOE-ε4+ and ε3ε3 and also between APOE-ε2+ and ε3ε3 groups.

#### Diffusion tensor imaging

The same participants compared in VBM were compared in DTI analysis. 2D Axial DTI images were acquired with a Siemens 3.0 T MR scanner with spin echo, echo planar imaging sequence, 54 gradient directions, a b-value of 1000 s/mm^2^, and voxel size of 2x2x2 mm^3^, 2mm slice thickness, 90^o^ flip angle, and a repetition time of 9600 ms.

Like in VBM preprocessing, images obtained from ADNI were first converted to *NIfTI* format. Next, eddy current correction (ECC) was performed to correct for image artifacts, such as tract distortions resulting from circulating currents in the gradient coils [30]. Following ECC, each participant’s brain was extracted, using the brain extraction tool (BET) in FSL, from the skull and neck and a binary brain mask was created [27]. Individual images were inspected in *FSLeyes* image viewer in order to optimize brain tissue inclusion by adjusting the fractional intensity threshold (thresholds ranged from 3.0–5.0). Finally, diffusion tensors were fit to every voxel using *DTIFIT*, outputting fractional anisotropy (FA) and mean diffusivity (MD) values for each participant to be included in a statistical comparison [31, 32].

#### Cognitive

Subjects included in the current study have undergone thorough baseline neuropsychological assessments. The cognitive measures used in the current study were comprised of composite scores for memory (ADNI-Mem) and executive functioning (ADNI-EF) derived from the ADNI neuropsychological test battery. Composite scores were determined by ADNI’s primary investigators using confirmatory factor analysis, representing multiple individual measures in each domain as an overall factor score [33, 34].

The ADNI-Mem composite includes the Rey Auditory Learning Test of episodic verbal memory (RAVLT; Trials 1–5, List B, Immediate Recall, Delayed Recall, and Recognition), Alzheimer’s Disease Assessment Scale-Cognitive memory-related measures (ADAS-Cog; Trials1-3, Recall, Recognition Present, Recognition Absent), Weschler Memory Scale Logical Memory I and II (WMS-R; Immediate and Delayed Recall), and the Mini Mental State Examination recall items (MMSE; ball, flag, & tree recall). The ADNI-EF composite includes the Wechsler Adult Intelligence Scale-Revised (WAIS-R; Digit Symbol Substitution), Digit Span Backwards, Trails A and B, Category Fluency (animals & vegetables), and Clock Drawing [34].

### Statistical analyses

#### VBM

Following preprocessing steps, Randomise permutation testing was used to compare GMV differences between the APOE ε4+ group and ε3ε3 controls and between the ε2+ group and ε3ε3 controls. Covariates of age, sex, and education were included in design matrices for both comparisons. Smoothing was applied, and comparisons were carried out using Randomise, which computes independent sample t-tests with 5000 permutations and corrects for multiple comparisons via TFCE to display any significant clusters in FSLeyes.

#### DTI

Following preprocessing steps, voxel-wise statistical analysis was performed using Track-based Spatial Statistics (TBSS) and Randomise to compute test statics in FSL [35, 36]. TBSS condenses all FA data into one standard tract skeleton to create average tracts across participants [37]. Individual FA values for each subject were registered onto a standard skeleton in 1x1x1 mm standard space (FMRIB59_FA), with each value projected onto a mean FA skeleton. Whole brain FA and MD values were compared between the APOE ε4+ group and ε3ε3 controls and then between the APOE ε2+ group and ε3ε3 controls. Covariates of age, sex, and education were included in design matrices for both comparisons. Comparisons underwent Randomise, which provided test statistics, and, finally, results corrected for TFCE were displayed in FSLeyes similarly to VBM.

#### MANCOVA and cognitive-structural neural correlates

In RStudio (Version 1.4.1103), a between-subjects multivariate analysis of covariance (MANCOVA) was performed across APOE groups with the two cognitive composite outcome measures and the inclusion of sex, age, and education level as covariates. Assumptions of univariate (using Shapiro-Wilk’s W) and multivariate normality were tested (using Henze-Zirkler’s HZ), as well as homogeneity of covariances (using Box’s M). Finally, Pillai’s Trace test statistic (*V*) was computed to test main effects in the current model. Effect sizes were determined by partial eta squared (η^2^).

The two composite scores were evaluated separately in each APOE group, regardless of significant between-groups differences in MANCOVA, to determine cognitive-neural correlates. Mean, whole-brain FA, MD, and GMV values were extracted from FSL for correlational analysis with cognitive neural correlates. In RStudio, Pearson correlations (r) were calculated for each composite score (i.e., ADNI-Mem & EF) and imaging metric (i.e., FA, MD, and GMV), then compared across groups visually via scatterplots.

## Results

### Participant demographic and cognitive characteristics

There were no significant differences between APOE groups in age, sex, or education. There were also no significant differences between groups on composite measures of memory and EF. Statistics for these data are reported in Table 1. Results from VBM and DTI analyses are presented in the following sections at significance (p<0.05, corrected for multiple comparisons via TFCE). The approach taken by Honea et al. examined uncorrected maps to examine and report trend-level patterns [7]. We took a similar approach in the current study; due to widespread, non-specific regions of difference, however, no meaningful patterns could be reported in the current study.

### VBM

VBM analyses revealed no significant differences in GMV between ε4+ and controls and between ε2+ and controls (p>0.05, corrected via TFCE).

### DTI

DTI analyses revealed no significant differences in FA or MD (p >0.05, corrected via TFCE) between ε4+ and controls, similarly between ε2+ and controls.

### MANCOVA and cognitive-structural neural correlates

**Fig 1:**
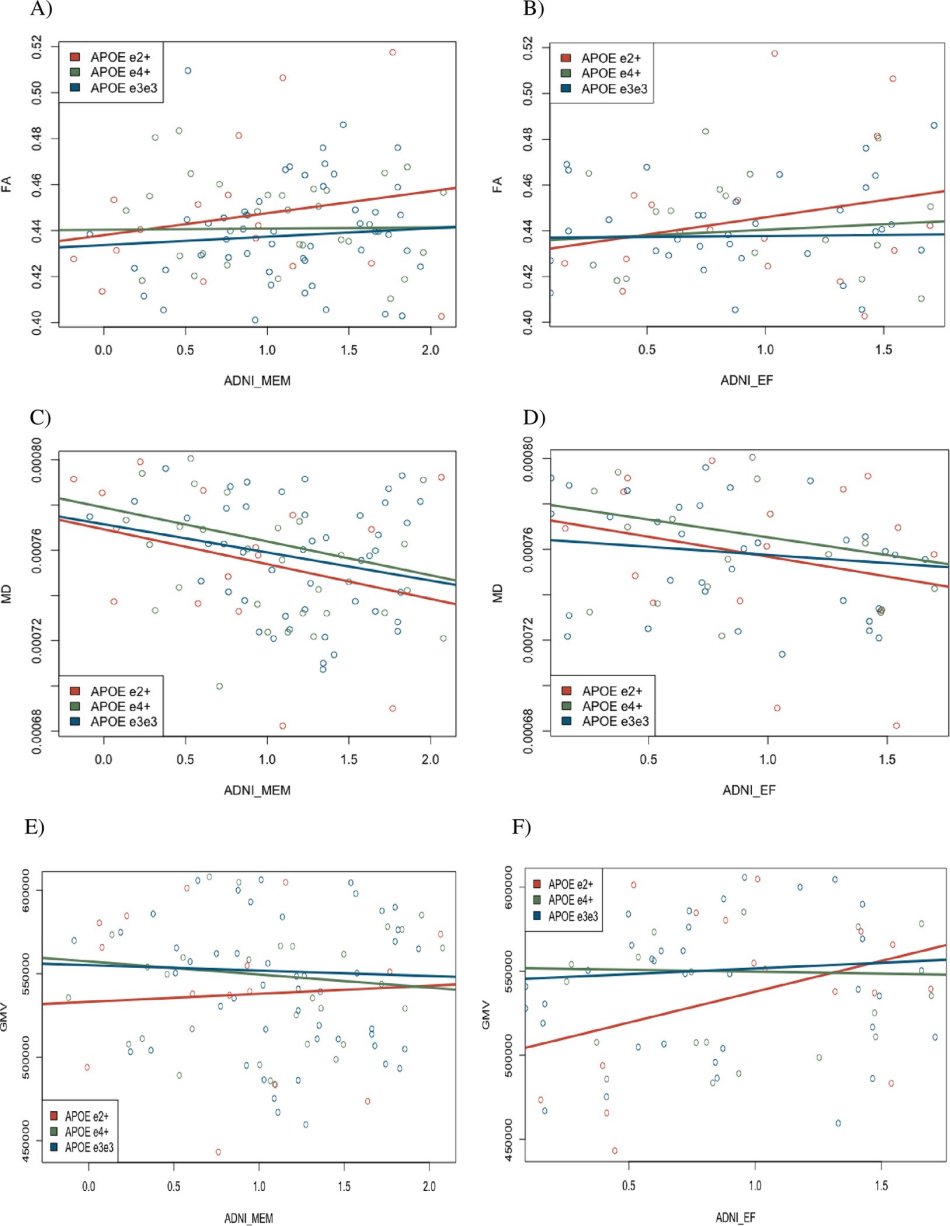
Scatterplots displaying within-group correlations for each APOE group. (A) FA x memory, (B) FA x EF, (C) MD x memory, (D) MD x EF, (E) GMV x memory, (F) GMV x EF.

For MANCOVA, assumptions were met for univariate normality (Memory: W = 0.99, p = 0.37; EF: W = 0.99, p-value = 0.69), multivariate normality (HZ = 0.46, p = 0.70), and homogeneity of covariances using (Box M, p = 0.27). There were no significant main effects of APOE status on cognitive outcome variables using Pillai’s trace (*V* = 0.06, F(4,206) = 1.51, p = 0.20). Effect sizes were low for memory scores (η^2^ = 0.035) and EF scores (η^2^ = 0.037) across groups. For within-group correlations, no significant relationships were found for any of the cognitive neural correlates (i.e., Fig 1A: FA x Memory, Fig 1B: FA x EF, Fig 1C: MD x Memory, Fig 1D: MD x EF, Fig 1E: GMV x Memory, & Fig 1F: GMV x EF) scores in APOE-ε4+, APOE-ε2+, or APOE-ε3ε3 controls. Table 2 displays non-significant correlational data within each APOE group.

**Table 2:** Correlational data for APOE groups.

Correlated Variables	APOE ε2+	p-value	APOE ε3ε3	p-value	APOEε4+	p-value
**FA x ADNI_MEM**	.20 [-.33, .63]	0.46	.08 [-.19, .34]	0.55	.01 [-.32, .34]	0.94
**FA x ADNI_EF**	.23 [-.30, .65]	0.39	.03 [-.24, .29]	0.82	.18 [-.16, .48]	0.30
**MD x ADNI_MEM**	-.29 [-.69, .24]	0.27	-.15 [-.40, .12]	0.27	-.23 [-.52, .11]	0.19
**MD x ADNI_EF**	-.25 [-.66, .28]	0.36	-.15 [-.40, .12]	0.28	-.33 [-.60, .01]	0.05
**GMV x ADNI_MEM**	.06 [-.45, .54]	0.82	-.04 [-.30, .23]	0.80	-.12 [-.44, .22]	0.48
**GMV x ADNI_EF**	.36 [-.17, .72]	0.18	.13 [-.14, .38]	0.36	-.05 [-.38, .29]	0.77

*Note*. Values represent Pearson correlation coefficients with lower and upper limits of the 95% confidence interval in brackets. *indicates *p* < .05 (no correlations showed significance).

## Discussion

The current manuscript aimed to replicate and expand upon a previous multimodal neuroimaging study conducted by Honea et al. [7], which examined the impact of APOE-ε4 presence on brain structure and cognitive function in healthy older adults. Using participants from the ADNI3, the current study followed closely Honea et al.’s multimodal imaging approach to compare APOE-ε3e3 individuals to APOE-ε4 carriers on GMV and WMI, with the addition of an APOE-ε2 carrier group and cognitive composite measures to expand on the previous findings [7]. The current study contributed to a growing body of literature investigating both the risk and protective effects of APOE-ε alleles (ε4 and ε2, respectively) on measures of cognitive performance, GMV and WMI in healthy older adults.

It was hypothesized that structural differences in GMV and WMI would be seen between APOE genotypes (i.e., APOE ε4-carriers <ε3ε3 controls < ε2-carriers) in a cohort of cognitively healthy older adults. Contrary to the hypothesis, no significant differences in grey or white matter were found between APOE-ε2 or APOE-ε4 carriers compared to APOE-ε3ε3 controls. These findings largely replicated Honea et al. [7], who similarly found no significant GMV differences between APOE-4 carriers and non-carriers. Although Honea et al. reported non-significant trend level differences between their APOE-e4 carrier and non-carrier groups in temporal, hippocampal, and frontal regions, the current study did not report non-significant findings [7].

Previous neuroimaging studies investigating APOE polymorphisms have found mixed results in cognitively healthy samples; thus, the current study aligns with some findings but contradicts others. Specifically, several studies have had similar null findings. Most recently, Lissaman et al. reported no significant differences in white or grey matter between healthy younger APOE-ε4 carriers and non-carriers, in line with the current study and previous work suggesting no differences in GMV between carriers and non-carriers of the ε4 allele [38–40]. Studies that have taken a longitudinal approach have also suggested that the presence of APOE-ε4 does not affect the rate of GMV change over time in the hippocampal or thalamic regions [22]. One notable limitation of these studies is that they did not include ε2-carriers and ε3ε3 controls separately, such as in the current study. In contrast, several other studies have demonstrated significant differences between APOE-e4 carrier and non-carrier groups. For example, Nao et al. found reduced GMV in APOE-ε4 carriers compared to non-carriers in prefrontal and temporal-parietal regions, as well as bilateral cingulate gyri [41], and den Heijer et al. found reduced GMV in the hippocampus of ε4 carriers compared to ε3ε3 controls [42]. In terms of WMI, Heise et al. found APOE-ε4 carriers had reduced FA compared to non-ε4 carriers [13], while Cavedo et al. found APOE-ε4 carriers showed increased MD and RD with decreased FA in several regions [43]. Findings additionally extend to early developmental stages, whereby grey and white matter development may be altered by APOE-ε4 presence and affect cognitive development as a result [44].

Within the aforementioned literature, studies varied by approach (multimodal or single modality comparison), age group (younger vs. older adults) and design (cross-sectional vs. longitudinal). Few studies have accounted for longitudinal trajectories. Interestingly, Haller et al. reported reduced GMV in the posterior cingulate cortex in APOE-ε4+ older adults who went on to develop subtle cognitive decline within 18 months but no differences in those who remained cognitively stable [20]. Another study found a significant impact of APOE-e4’s presence on hippocampal volume longitudinally only in people who later went on to develop MCI or AD but did not impact cognition, GMV, or WMI differently across healthy APOE groups cross-sectionally [45]. It is possible that the participants in the current study represent a mix of individuals; some later go on to develop cognitive decline, and others who remain cognitively stable over time. Longitudinal follow-up may become possible as ADNI data collection accumulates over time. Taken together, differences in methodological approaches and in the trajectories of healthy participants may account for the range of findings in the extant literature.

The scope of the current replication was broadened by comparing APOE-ε2 carriers and APOE-ε3e3 controls on GMV and WMI. Contrary to the study’s hypotheses, no significant differences between APOE-ε2 carriers and APOE-ε3e3 controls on GMV and WMI. Similar results have been reported in previous literature. Luo et al. found no differences in GMV or thickness across APOE-ε2 carriers, ε4 carriers, and ε3ε3 controls using data from ADNI healthy cohorts ADNI-GO and ADNI1 [19]. The current study has essentially replicated these GMV results in the ADNI3 cohort. Nonetheless, some previous studies have reported APOE-e2 changes to WMI and GMV. For example, Chiang et al. found an increase in ε2 homozygotes [46], and Konishi et al. found the ε2 allele to be associated with higher GMV in hippocampal and medial temporal regions compared to non-carriers, although their cognitively healthy sample was of much younger age than the current study [21]. Given that limited studies have focused on the potential neuroprotective effects of APOE-e2, further research on this group is needed.

Cognitive scores recorded from the healthy older cohort in the current study were expected to fall within the normal range on standardized measures; nonetheless, some previous studies have reported a relative cognitive advantage in ε2-carriers and a relative disadvantage in ε4-carriers [5, 7, 8–13, 46]. The current study found no significant differences between APOE groups on indices of memory or EF. In contrast, Honea et al. found APOE-ε4 carries had overall decreased global cognitive performance, with specific reductions on memory measures (i.e., Logical Memory I) and working memory (i.e., Letter-Number Sequencing) [7]. The current study did not replicate these findings, although the measures used to evaluate memory and EF in the current study varied slightly, which may explain the discrepant findings. The current study also evaluated cognitive-neural correlates (in line with data availability from ADNI3), while Honea et al. evaluated cognitive-lifestyle correlates [7]. The current findings did not detect significant relationships between cognitive scores and imaging metrics in any APOE groups.

### Limitations

While the current study followed Honea and colleagues’ methods as closely as possible, the measures selected in the current study were limited to those available in the ADNI [7]. For instance, ADNI does not include lifestyle and physical performance measures; therefore, cognitive neural correlates were evaluated similarly to Honea et al. evaluated lifestyle measures [7]. Nonetheless, ADNI addresses many other imaging and neuropsychological research limitations, including increasing replicability, sample size, and cost-effectiveness.

While the current healthy cohort was comparable to the replicated study (free of subjective memory complaints, exhibited normal memory function on the Logical Memory II, MMSE score between 24 and 30 inclusive, and a Clinical Dementia Rating of 0), there remained some overall issues with the approach to evaluating “healthy” in the ADNI. Criteria for this group were dependent on a select few criteria (i.e., WMS-R LMII, MMSE, & CDR scale) rather than a comprehensive neuropsychological examination. While effective in its quick ability to categorize ADNI participants, this leaves out certain cognitive domains which might show poor performance in this healthy sample, such as working memory and other EFs. The ongoing challenge with operationalizing healthy ageing lies in the various and limited approaches to assessment and inconclusive theoretical definitions applied in different ways throughout the literature.

The current study also differed from the approach employed by Honea and colleagues in terms of the software used for the VBM analysis and the conservativeness of the thresholds employed [7]. Specifically, the current study used FSL software for the VBM analysis with correction for multiple comparisons. In contrast, Honea et al. used SPM software and detected regional differences between groups in the uncorrected data [7]. Although the processing pipelines are similar, it is possible that variations in findings could relate to differences in the data analysis software [47]. Future replication studies should further examine the role that analysis software plays in variability in findings across the literature.

Furthermore, neither Honea and colleagues nor the current study employed an analysis approach to examine the relationships between lifestyle and cognitive variables within an imaging pipeline [7]. This approach could potentially identify specific regions that relate to these variables of interest. Future studies could expand on current findings by incorporating these variables into VBM and TBSS design matrices.

The sample size in the current study overall is comparable to other imaging studies, especially multimodal ones; however, sample size in imaging studies continues to pose limitations. The APOE-ε2 group in the current study is particularly small, although this was expected based on population prevalence rates. Nevertheless, it is possible that a larger sample size and greater power would have made it possible to detect subtle cognitive and neurostructural changes across these groups.

### Future directions

Future studies should continue to take a multimodal approach, and to be as comprehensive as possible. In particular, future studies could include additional MRI scan acquisitions (e.g., functional MRI) and analyses to expand on the current work focused on grey matter morphometry and white matter microstructure. Additional imaging metrics may allow future research to identify structural changes in healthy older adults with different APOE-ε alleles that may not have been detectable in the current study. Such metrics could include the additional DTI metrics of RD and AxD, alternative measures of grey matter morphology (e.g., cortical thickness analyses), and qMRI. Using these approaches, future studies may more readily identify key missing components to APOE’s complex neuromechanisms. For instance, a recent study looked at myelin water fraction using qMRI and found that healthy adult APOE-ε2 carriers had higher myelin content than APOE-ε4 carriers [48].

A longitudinal approach should also be taken in future studies. Several previous studies have shown differences in the rate of decline in both brain structure and function across APOE carriers. This type of design is particularly facilitated by large databases, like the ADNI, that continuously add participants with repeated assessments over time.

Finally, previous studies, including Honea et al., have most often examined APOE-ε4 carriers versus non-carriers [6, 7]. Future studies should separate APOE-ε3e3 from APOE-ε2 carriers when possible. Although the current study did not find differences between APOE-ε3e3 and APOE-ε2 carriers, further investigations on whether these groups have differing effects on neuropathology are required. Some studies also posit a gene-dose effect, with the addition of each ε4 risk allele increasing pathology severity, and the opposite for the ε2 allele (e.g., in Hostage et al.) [49]. Future studies should include homozygote ε2 and ε4 groups to examine these gene-dose effects.

## Conclusion

The current study replicated the main findings of Honea and colleagues, which revealed no significant differences in WMI or GMV between healthy older adults with and without the APOE-ε4 allele [7]. The current study also aimed to extend previous findings by examining the APOE-ε2 allele, which is thought to confer some protective effects against neurodegeneration. Based on the current study and other mixed findings to date, whether these effects occur through the preservation of grey and white matter structure is unclear. The current and replicated study shows that different APOE-ε isoforms do not differentially impact brain structure and function in older adults free of cognitive impairment. Future research should examine large sample sizes and multimodal measures over multiple time points to understand the possible trajectories of healthy ageing that may emerge as a function of APOE alleles.

## Supporting information

S1 Checklist STROBE statement—checklist of items that should be included in reports of observational studies. (DOCX)
